# Rehabilitation modalities in ankylosing spondylitis: orthopaedic perspectives on function, bone health, and surgical safety

**DOI:** 10.1016/j.jor.2026.03.031

**Published:** 2026-03-20

**Authors:** Naveen Jeyaraman, Swaminathan Ramasubramanian, Aadithya Siddarth Sridhar, Arulkumar Nallakumarasamy, Sathish Muthu, Luise Schäfer, Filippo Migliorini, Madhan Jeyaraman

**Affiliations:** a Department of Regenerative Medicine, Agathisha Institute of Stem Cell and Regenerative Medicine (AISRM), Chennai, Tamil Nadu, 600030, India; b Department of Orthopaedics, ACS Medical College and Hospital, Dr MGR Educational and Research Institute, Chennai, Tamil Nadu, 600077, India; c Department of Orthopaedics, Orthopaedic Research Group, Coimbatore, Tamil Nadu, 641045, India; d Department of Orthopaedics, Faculty of Medicine – Sri Lalithambigai Medical College and Hospital, Dr MGR Educational and Research Institute, Chennai, Tamil Nadu, 600095, India; e Department of Orthopaedics, Jawaharlal Institute of Postgraduate Medical Education and Research (JIPMER)-KARAIKAL, Puducherry, 609602, India; f Central Research Laboratory, Meenakshi Medical College Hospital and Research Institute, Meenakshi Academy of Higher Education and Research, Chennai, Tamil Nadu, 631552, India; g Department of Trauma and Reconstructive Surgery, University Hospital of Halle, Martin Luther University Halle-Wittenberg, 06097, Halle (Saale), Germany; h Department of Orthopaedic and Trauma Surgery, Eifelklinik St. Brigida, Kammerbruchstr. 8, 52152, Simmerath, Germany; i Department of Life Sciences, Health, and Health Professions, Link Campus University, Via del Casale di San Pio V, 00165, Rome, Italy

**Keywords:** Ankylosing spondylitis, Axial skeleton, Telerehabilitation, Physical rehabilitation

## Abstract

Ankylosing Spondylitis (AS) is a chronic inflammatory disorder primarily affecting the axial skeleton, leading to progressive spinal fusion, kyphotic deformity, and heightened fracture risk. This review synthesises current evidence on physical rehabilitation strategies essential for orthopaedic management across disease stages. Key assessment tools—Bath Ankylosing Spondylitis Disease Activity Index (BASDAI), Bath Ankylosing Spondylitis Functional Index (BASFI), and Bath Ankylosing Spondylitis Metrology Index (BASMI)—enable multidimensional evaluation of disease activity, functional capacity, and spinal mobility. Conservative rehabilitation, particularly land-based exercise (LBE) and aquatic therapy, demonstrates moderate efficacy in improving mobility and function, with aquatic modalities offering superior pain relief and psychological benefits. Supervised physical therapy outperforms unsupervised programs, leading to greater adherence and better clinical outcomes. Exercise also contributes to bone mineral density (BMD) enhancement, especially at the femoral neck and hip, with mind-body and resistance training ranking highest in efficacy. However, current protocols have a limited impact on pathological bone formation (ankylosis), underscoring the need for optimised mechanical loading strategies. Telerehabilitation offers accessibility benefits but may lack the fidelity of in-person supervision, especially for complex biomechanical corrections. Perioperative rehabilitation is critical in surgical candidates, with prehabilitation targeting metabolic optimisation and postoperative protocols tailored to protect instrumentation and restore function. Total Hip Arthroplasty (THA) requires strict adherence to positioning precautions due to spinal rigidity, and prophylaxis against heterotopic ossification is mandatory. Overall, physical rehabilitation is indispensable in mitigating orthopaedic complications, preserving mobility, and enhancing quality of life in AS patients. High-fidelity, supervised, and multimodal programs should be prioritised to maximise therapeutic outcomes and delay surgical intervention.

## 1 Introduction

Ankylosing Spondylitis (AS) is a chronic inflammatory disease of unknown aetiology, primarily affecting the axial skeleton.^1^ The condition is characterised by inflammation and progressive fusion of the sacroiliac joints, spine, and hips.^1^ Globally, the prevalence is approximately 0.9%, impacting an estimated 350,000 persons in the United States and 600,000 in Europe, predominantly Caucasian males, often commencing between the second and fourth decades of life. Genetic linkage to the human leukocyte antigen B27 (HLA-B27) has been well established.^2^

The pathophysiological progression involves chronic inflammation leading to new bone formation in the spine, resulting in progressive fusion, known as ankylosis, which severely restricts mobility over time.^3^ This structural damage results in severe functional impairment in roughly 30% of diagnosed patients. Advanced disease states frequently manifest as dorsal hyperkyphosis and the abolition of lumbar lordosis, creating characteristic postural alterations that compromise gait and visual fields.^4^ The consequence of spinal ankylosis is the transformation of a flexible, load-bearing structure into a rigid, brittle column highly susceptible to fracture. Spinal fractures in AS patients often occur following minor trauma and are frequently missed, necessitating thorough diagnostic assessment beyond plain radiographs.^5^

The risk profile for these complications is quantitatively significant. A retrospective, primary care-based nested case-control study utilised data from the UK General Practice Research Database, including 758 AS patients.^6^ This study determined that the risk of clinical vertebral fractures (VFs) was increased by an odds ratio (OR) of 3.3 compared with subjects without AS, even after adjustment for relevant confounders.^7^ Furthermore, a Swedish retrospective case-control hospital discharge study involving 265 AS patients reported that the risk of all fractures was increased (n = 181, OR: 4.0), and specifically, the risk of hip fractures was increased (n = 64, OR: 2.5).^8^

Fracture management in AS represents a high-stakes orthopaedic challenge. Analysis of the National Inpatient Sample (NIS) data (2016–2018) involving 5385 patients demonstrated that AS patients with spinal fractures have significantly higher postoperative complications than the general population. The overall complication rate recorded was 40.8**%**, with respiratory complications, including pneumonia and respiratory insufficiency, as the predominant pattern.^5^ The presence of a spinal cord injury (SCI) increased the odds of having a complication by 2.164 times (95% confidence interval, 1.722–2.72; p < 0.001). This exceptionally high complication rate mandates that the orthopaedic approach must aggressively prioritise fracture prevention through conservative physical management as a core strategy for risk mitigation.^9^

Standardised outcome measures, validated by international consensus groups, are essential for evaluating the efficacy of physical rehabilitation protocols. The Bath Ankylosing Spondylitis indices serve as the principal instruments^10^:

● **Bath Ankylosing Spondylitis Disease Activity Index (BASDAI):** This six-question instrument assesses subjective symptom severity over the past week, including fatigue, spinal pain, joint pain/swelling, localised tenderness, and the duration of morning stiffness.^11^ A higher score indicates greater disease activity, which correlates with the inflammatory burden driving structural damage.^12^
● **Bath Ankylosing Spondylitis Functional Index (BASFI):** This 10-item scale evaluates the degree of functional limitation in performing daily activities. A high score reflects greater functional impairment, often serving as a key indicator of disability and the potential necessity for major orthopaedic interventions like arthroplasty or osteotomy.^13^
● **Bath Ankylosing Spondylitis Metrology Index (BASMI):** The BASMI is the most widely reported, validated, objective measure of axial mobility.^14^ It assesses mobility across five measurements: the modified Schober's test (lumbar flexion), tragus-wall distance (cervical extension/kyphosis), cervical rotation, lumbar lateral flexion, and intermalleolar distance. High scores denote severe limitations, reflecting the irreversible progression of bony fusion (ankylosis).^15^

The need to target simultaneous improvement across all three BAS indices—activity (BASDAI), function (BASFI), and objective mobility (BASMI)—is clear (Table 1).^16^ Relying solely on pain reduction via pharmacological management without achieving concomitant objective mobility gains risks masking ongoing, irreversible structural damage (ankylosis) that is the precursor to complex kyphotic deformity and acute spinal fracture.^17^

**Table 1:** Key assessment indices in ankylosing spondylitis rehabilitation.

Index	Domain Assessed	Scoring Interpretation	Orthopaedic Relevance
BASDAI	Disease Activity (Pain, Stiffness, Fatigue)	Higher score = Greater disease activity (0–10)	Correlates with inflammation driving structural damage and fracture risk.
BASFI	Functional Capacity (Activities of Daily Living)	Higher score = Greater functional limitation (0–10)	Direct measure of physical disability and surgical necessity assessment.
BASMI	Spinal Mobility (Cervical, Lumbar, Intermalleolar)	Higher score = Severer limitations of movement (0–10)	Objective measure of progressive bony fusion (ankylosis) and postural risk.

## 2 Conservative rehabilitation: efficacy of modality types and delivery

The cornerstone of non-pharmacological management for axial Spondyloarthritis (axSpA) is physical rehabilitation, as stipulated by the 2022 Assessment of SpondyloArthritis International Society (ASAS)/European Alliance of Associations for Rheumatology (EULAR) recommendations.^18^

### 2.1 Land-based exercise (LBE) programs: components and quantitative outcomes

LBE programs are essential for mitigating mobility and functional loss in AS. A standard LBE regimen integrates flexibility, strengthening, and aerobic components.^19^ Flexibility exercises, including range-of-motion work, are critical to prevent joint stiffness; strengthening exercises, specifically targeting neck and abdominal muscles, help improve posture, reduce pain, and counteract the tendency toward forward bending.^20^

Overall, exercise programs, regardless of the specific type, demonstrate a statistically moderate effect on disease activity, function, and spinal mobility.^21^ However, specialised combinations yield better results for specific domains. Programs that combine flexibility and muscle-strength exercises show the largest effect on spinal mobility (BASMI).^20^ Furthermore, including aerobic exercise demonstrates significant efficacy for improving function (BASFI). While aerobic training improves walking distance and capacity, some data suggest it does not provide additional benefits in functional capacity or mobility compared with stretching exercises alone.^22^

Despite these observed benefits, the measured improvements in spinal mobility often remain small in magnitude, with effect sizes (ES) ranging from 0.02 to 0.67 reported across trials.^23^ A Cochrane review identified moderate-to low-quality evidence suggesting that exercise programs may have little or no clinically meaningful effect on improving function or reducing pain compared with usual care.^24^ This finding highlights a crucial distinction: while exercise is biologically effective, the often small quantitative gains in objective mobility (BASMI) underscore the profound difficulty in reversing established axial rigidity.^25^ Given that kyphotic deformity often precedes complex spinal osteotomy, maximising axial mobility through optimal, high-adherence programs is a primary therapeutic goal to prevent progression toward major surgery.^26^

### 2.2 Aquatic exercise (WBE) and balneotherapy: biomechanical advantages and efficacy

Aquatic exercise leverages the physical properties of water, specifically buoyancy and resistance, to enable less painful movement and muscle relaxation compared to land-based activities.^27^ A randomised controlled trial (RCT) involving 69 AS patients compared a 4-week aquatic exercise protocol (20 sessions, 5 times per week, in 32-33 °C water) with a home-based LBE program (a single physiotherapist demonstration followed by manual provision). Although both groups showed significant improvements across multiple indices (p < 0.05), the aquatic exercise group demonstrated significantly greater improvements at both the 4-week and 12-week follow-ups relative to pretreatment values.^28^ The aquatic exercise group achieved superior reductions in pain (Visual Analogue Scale, VAS) (p < 0.001) and provided greater psychological and functional benefits across multiple subcomponents of the SF-36, including bodily pain, general health, vitality, social functioning, role limitations due to emotional problems, and general mental health.^29^ The magnitude of the superiority for pain reduction (p < 0.001) supports the integration of aquatic therapy as a powerful analgesic and initial mobility enhancer, especially in patients with high baseline pain that restricts participation in LBE.^30^

Balneotherapy, often involving bathing in natural thermal or mineral waters, is also effective. Studies have reported statistically significant improvements in BASDAI, BASFI, BASMI, and the Ankylosing Spondylitis Disease Activity Score-C-reactive protein (ASDAS-CRP) scores across treatment groups at both 4 and 12 weeks of follow-up (p < 0.001).^31^ The therapeutic benefits are attributed to biological mechanisms, including reductions in circulating levels of pro-inflammatory mediators such as prostaglandin E2 (PGE2), leukotriene B4 (LTB4), interleukin-1beta (IL-1β), and tumour necrosis factor-alpha (TNF-α).^32^

### 2.3 Supervised vs. unsupervised (home-based) programs

The fidelity and supervision method significantly affect clinical outcomes and adherence. Supervised exercise or physical therapy is consistently recommended as more clinically suitable than unsupervised home programs.^33^ Supervised combined exercise or neuromuscular training provides greater gains in symptom severity (BASDAI), physical function (BASFI), and spinal flexibility (BASMI) compared to standard care.^34^

A meta-analysis comparing specific exercise modalities (Pilates, aquatic, Global Postural Re-education) with conventional physical therapy found that, overall, the outcomes for BASMI, BASDAI, and BASFI favored physical therapy.^35^ Specific exercises generally showed a small to moderate effect size on impairment and activity limitations, whereas physical therapy outcomes exhibited slightly higher efficacy across these core indices.^36^ This observation suggests that the critical factor is not necessarily the specific exercise type (e.g., Pilates) but rather the consistency, expert-intensity control, and professional corrective feedback provided through high-fidelity, supervised physical therapy.^37^

From an economic perspective, supervised physical therapy is generally cost-effective. A cost-effectiveness analysis comparing supervised group physical therapy to unsupervised home exercises in AS patients found that, while group therapy incurred additional costs of $531 per patient per year, it also reduced direct medical costs by $122 per year. This resulted in a net cost of $409 per patient per year for the beneficial effects of supervised group therapy.^38^ This relatively minor cost supports the widespread implementation of supervised programs, especially considering the substantial financial burden associated with managing major orthopaedic complications like spinal fractures.^39^ The efficacy of exercise modalities on ankylosing spondylitis outcomes is tabulated in Table 2.

**Table 2:** Comparative efficacy of exercise modalities on core as outcomes.

Intervention Type	Effect Size (ES/SMD) on Impairment/Activity Limitation		Outcomes Favoring Intervention	Clinical Detail/Follow-up	Source
Land-Based Exercise (LBE) Combined Strength/Flexibility	Moderate Effect (General)	BASMI (Largest effect on spinal mobility)	Spinal mobility ES range: 0.02–0.67 (Small improvements reported).	15	
Specific Exercises (e.g., Pilates, GPR)	Small to Moderate Effect	Chest Expansion, Pulmonary Function	BASMI, BASDAI, BASFI favored conventional physical therapy comparator.	24	
Aquatic Exercise (WBE) vs. Home LBE	Significantly Superior Efficacy	VAS, Bodily Pain, General Health ($p < 0.001$ for all)	WBE protocols (20 sessions, 32-33 °C) yielded sustained psychological and pain benefits up to 12 weeks.	20	
Supervised Group Therapy vs. Home Exercise	Superior Clinical Benefit	BASDAI, BASFI, BASMI improvements	Net cost difference: $409 per patient per year (Group cost $531/yr, offset by $122/yr reduced medical costs).	25	

## 3 Rehabilitation for structural integrity and bone metabolism

The management of bone mineral density (BMD) and the pathological formation of new bone (syndesmophytes) are critical orthopaedic concerns driven by the AS disease process. Patients suffering from AS are at an increased risk of developing thinning of the bones (osteoporosis) and subsequent spinal fractures.^40^ The prevalence of low BMD is particularly high among elderly patients with AS.^41^

Orthopaedic assessment of osteoporosis in AS requires modification due to the inflammatory bone changes.^42^ Traditional Dual-energy X-ray Absorptiometry (DEXA) measurements at the lumbar spine (LS) can be artifactually elevated due to vertebral sclerosis and syndesmophyte formation, leading to potential misdiagnosis or underestimation of fracture risk.^43^ Therefore, quantification of BMD at the femoral neck (FN) is considered a more reliable and effective metric for evaluating true osteoporotic status in AS patients.^44^

Exercise intervention has been shown to positively influence BMD. A systematic review and meta-analysis reported a statistically significant (p < 0.001), but rather low, effect (Standardised Mean Difference, SMD = 0.33–0.40) of exercise on BMD at the LS and proximal femur.^45^ This small effect size is often attributed to heterogeneity in protocols and to the inclusion of studies with potentially inadequate exercise regimens in terms of dosage and intensity.^46^

To define optimal exercise modalities for managing BMD, Network Meta-Analysis (NMA) studies have provided clearer direction^47^:

● **Total Hip (TH) and FN Density:** Progressive exercise training programs have shown notably superior outcomes compared to control groups. One study reported an SMD of 1.54 (95% CI 0.76–2.33, p < 0.001) for FN BMD and an SMD of 1.34 (95% CI 0.41–2.28, p < 0.001) for total hip BMD.^48^
● **Optimal Modalities (NMA ranking):** Mind-body exercise was identified as the optimal exercise type for increasing FN BMD (SUCRA = 0.99), while resistance exercise was the most promising type for total hip BMD (SUCRA = 0.95).^49^

The implication for orthopaedic prehabilitation is that exercise protocols must prioritise high-intensity, progressive resistance training and mind-body exercises targeting the appendicular skeleton.^50^ Since spinal fracture risk correlates strongly with hip fracture risk, maximising hip BMD via these modalities is one of the most actionable conservative strategies for systemic fracture prevention.^51^

### 3.1 Exercise and the biomechanics of ankylosis: the sclerostin hypothesis

**Fig. 1:**
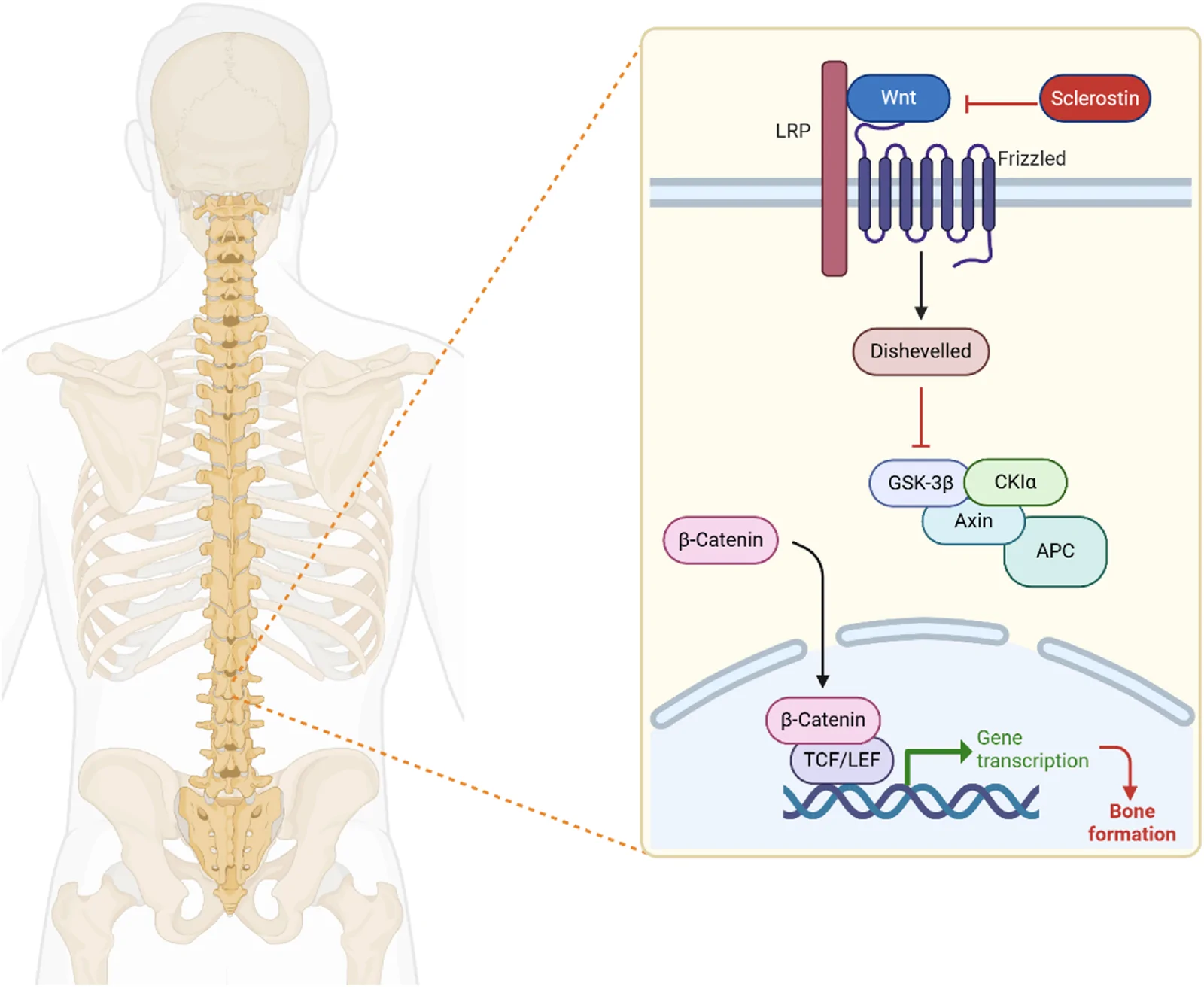
Role of Sclerostin in the pathophysiology of syndesmophyte formation.

The pathological formation of syndesmophytes is the primary determinant of long-term disability and rigidity in AS.^32^ New bone formation is regulated by the Wnt-β-catenin signalling pathway, as shown in Fig. 1.^52^ Low serum sclerostin levels (SSLs), in which sclerostin is an inhibitor of this pathway, have been associated with new syndesmophyte formation and subsequent radiographic progression.^53^

In healthy individuals, mechanical loading via exercise is known to influence bone remodeling.^54^ However, the role of therapeutic exercise in modulating the pathological bone formation associated with AS remains unclear and poses a biological challenge, as animal models suggest that mechanical stress may contribute to entheseal inflammation and new bone formation.^55^

A study comparing Balneotherapy, Water-Based Exercise (WBE), and Land-Based Exercise (LBE) investigated the effect of these treatments on SSL in AS patients (n = 60).^35^ While all three groups achieved statistically significant improvements in clinical parameters (BASDAI, BASFI, BASMI, p < 0.05), the changes in SSL were not statistically significant in any group (p > 0.05) at 4 or 12 weeks of follow-up.^56^

This lack of detectable biochemical change suggests that current standardised therapeutic exercise protocols, while effective for functional maintenance and pain reduction, may not provide sufficient mechanical stimulus to alter the underlying anabolic-catabolic imbalance driving structural disease progression (ankylosis) in a measurable way.^57^ This highlights a critical research gap: determining the optimal high-intensity mechanical loading necessary to definitively influence the pathological bone formation cascade, or, conversely, establishing the intensity threshold at which exercise may become detrimental due to exacerbating entheseal stress.^52^

### 3.2 Perioperative physical rehabilitation in orthopaedic AS management

Surgical intervention in advanced AS, typically involving corrective spinal osteotomy (for kyphosis) or Total Hip Arthroplasty (THA, for severe hip involvement in 25–50% of young-onset cases), is complex and carries exceptionally high risk. Rehabilitation serves a critical protective function in the perioperative period.^58^

#### 3.2.1 Prehabilitation

Prehabilitation is a comprehensive, multidisciplinary preoperative process intended to reduce perioperative complications, pain, postoperative disability, and the length of hospital stay (LOS).^59^ Given the high complication rate of AS surgery, adherence to rigorous metabolic and physical prehabilitation targets is non-negotiable.^60^ Preoperative screening and optimisation are vital to mitigate specific AS-related surgical risks, such as poor wound healing, infection, and hardware failure due to compromised bone quality.^61^ Specific metabolic goals are summarised in Table 3.

**Table 3:** Preoperative optimisation targets for as patients undergoing spine or hip surgery.

Risk Factor/Parameter	Preoperative Goal/Target Value	Intervention Relevance	Cited Risk Mitigation	Source
Hemoglobin (Hb)	>12 g/dL (Women), >13 g/dL (Men)	Hematology consult, anemia workup.	Reduced mortality rate, infections, and complications.	44
Vitamin D (25OHD)	>30 ng/mL	Initiate supplementation (e.g., $50,000$ IU weekly).	Optimisation for bone remodeling, fusion stability, and fracture prevention.	44
Bone Mineral Density (BMD)	T-score > −2.5 Ideally > −1.0	Endocrinology referral, anti-resorptive/anabolic therapy consideration.	Mitigate high risk of vertebral fractures and difficulty with instrumentation.	5
Opioid Use	Wean to complete cessation (6–8 weeks before surgery)	Pain management referral, possible behavioral therapy.	Reduction in postoperative complication rate and LOS.	44
Hyperglycemia/A1C	Tight control, proactive basal/bolus insulin management.	Endocrinology consult.	Minimize risk of surgical site infections and overall complication profile.	45

Functional conditioning, including formal physical therapy or aquatic therapy, should be maintained until three days before surgery to sustain cardiopulmonary fitness and core stability.^62^ Furthermore, behavioral adjustments, such as smoking cessation initiated at least one month prior to elective surgery, are mandatory to minimize complications.^63^

#### 3.2.2 Postoperative management

Spinal corrective osteotomy, such as Pedicle Subtraction Osteotomy (PSO) or Vertebral Column Decancellation (VCD), is performed to correct severe thoracolumbar kyphosis.^64^ The complication profile is dictated by the rigidity of the spine and the invasiveness of the procedure. Correction using a one-level osteotomy has a complication rate of 6.5%, whereas a two-level osteotomy increases the rate significantly to 23.6%.^65^ Furthermore, historic cohort studies report high incidences of specific complications, including permanent neurologic deficits (7.8%), deep wound infections (9.6%), and major general complications (10.4%).^66^

Postoperative physical therapy is phased to protect the instrumentation and maintain fusion stability.^67^

● **Acute Phase (Weeks 1**–**4):** This phase focuses on early mobilisation within surgical restrictions. Key milestones include walking for 15–30 min several times daily, performing gentle physical therapy exercises, and transitioning to over-the-counter pain management.^68^ Strict postural control and core muscle activation are emphasised to protect the spine.^69^
● **Intermediate Phase (Weeks 6**–**8):** Physical therapy intensity increases, focusing on specific exercises to improve core stability and strengthen surrounding muscles, tailored to the specific osteotomy site and fusion goals.^70^
● **Advanced Phase (Weeks 9**–**12):** Progression includes resuming most work responsibilities, participating in low-impact recreational activities, and performing more complex exercises to ensure durable functional recovery.^71^

Total Hip Arthroplasty (THA) is the most effective intervention for relieving pain and restoring function in AS patients with advanced hip disease.^72^ However, AS patients face a recognised risk of peri-implant Heterotopic Ossification (HO).^73^ Prophylaxis is mandatory for HO prevention. Both non-steroidal anti-inflammatory drugs (NSAIDs) and low-dose radiotherapy are widely accepted prophylactic options.^74^

● **Radiotherapy Protocol:** Radiotherapy is highly effective, typically delivered as a single dose of 7 Gy to 8 Gy, administered either pre-operatively (less than 4 h before surgery) or post-operatively (within 72 h of surgery).^75^
● **Pharmacological Protocol:** NSAIDs, particularly selective cyclooxygenase 2 (COX-2) inhibitors, are frequently used due to lower cost and ease of administration. A regimen often used involves 60 mg etoricoxib daily for two weeks.^76^ The orthopaedic team must select the appropriate prophylactic strategy based on patient risk and comorbidities.^77^

Long-term monitoring for HO is necessary, often utilising the Brooker classification system on follow-up radiographs, as HO can occur or increase several years postoperatively due to new trauma or implant loosening.^78^ Postoperative physical therapy begins immediately, focusing on improving circulation and initiating gentle range-of-motion exercises (e.g., ankle pumps, ankle rotations).^79^ The rigidity of the fused AS spine complicates post-THA positioning, as the rigid lumbar segment transfers greater rotational and flexion stress to the new hip joint capsule.^80^ Therefore, standard THA precautions must be strictly enforced:

1. Avoid bending the new hip more than 90°.^81^
2. Avoid bending forward more than 90° (e.g., difficulty putting on socks and shoes).^81^
3. Avoid crossing the operated leg over the other leg.^81^
4. Avoid turning the operated leg inward.^81^

The acute-care phase emphasises safe transfers and protected ambulation with assistive devices.^82^ Subsequent phases focus on progressively restoring the strength of the proximal hip musculature (gluteals, iliopsoas) and core stability, while strictly maintaining hip precautions until functional demands are met.^83^

### 3.3 Clinical recommendations for orthopaedic practice

Physical rehabilitation is fundamentally an indispensable tool for orthopaedic risk management and functional preservation in Ankylosing Spondylitis. Evidence strongly supports the necessity of non-pharmacological management in combination with medical therapy across all disease stages.^84^

The overall quantitative evidence demonstrates that supervised physical therapy is clinically superior to unsupervised home-based programs, offering greater improvements in objective mobility (BASMI), function (BASFI), and disease activity (BASDAI).^85^ This superiority is achieved at a manageable net cost of $409 per patient per year. Aquatic exercise provides unique benefits, achieving superior pain reduction (VAS, p < 0.001) compared with home exercise, making it ideal for initiating therapy in highly symptomatic patients.^86^ To mitigate the systemic risk of fracture, orthopaedic rehabilitation must prioritise BMD improvement in the appendicular skeleton, given the unreliability of LS BMD readings in AS.^87^ Protocols should integrate progressive resistance training and mind-body exercises, which demonstrate the largest effects on total hip (SMD 1.34) and femoral neck BMD (SMD 1.54).^87^

For patients requiring surgical intervention (THA or spinal osteotomy), prehabilitation is a crucial safety measure.^88^ Orthopaedic teams must mandate strict adherence to metabolic and functional prehabilitation targets, including achieving a minimum Vitamin D level of >30 ng/mL and Hb levels of >12 g/dL (women) or >13 g/dL (men) to mitigate the high surgical complication risk (up to 40.8% post-fracture).^89^ Postoperatively, specialised prophylaxis against Heterotopic Ossification (7 Gy to 8 Gy radiation or NSAIDs) is essential after THA.^90^

Current physical rehabilitation programs, despite achieving functional benefits, do not appear to significantly modulate the specific biological pathways driving structural ankylosis, as evidenced by the statistically non-significant changes in serum sclerostin levels (p > 0.05).^91^ Future research must shift focus beyond functional metrics toward establishing the optimal high-intensity mechanical loading parameters necessary to structurally influence bone formation pathways without exacerbating inflammation, thereby addressing the long-term progression of bony fusion itself.^92^

### 3.4 Advanced delivery, adherence, and cost-effectiveness

#### 3.4.1 Barriers to long-term adherence

For rehabilitation to successfully mitigate the orthopaedic risks of AS, long-term patient adherence to prescribed exercise programs is mandatory. However, several disease-specific factors impede consistency. Primary patient-reported barriers to exercise include pain, stiffness, fatigue, disability, and poor sleep quality.^93^

Supervised exercise has proven moderately effective in increasing physical activity levels (PAL) and adherence. Studies have reported high adherence rates in supervised settings, with patients completing approximately 75% of sessions over a 12-week period.^94^ Furthermore, patients participating in supervised therapy sessions spent significantly longer on their mandatory home exercise program (HEP) compared to those without supervision (mean duration: 1.9 h versus 1.2 h per week, p < 0.05). This suggests that supervision acts as a functional catalyst, improving motivation and increasing the fidelity of the patient's long-term commitment.^95^ Given that pain and fatigue are significant barriers, early integration of modalities known to alleviate these symptoms (e.g., aquatic therapy for pain, Qigong for fatigue) is a necessary prerequisite for sustaining long-term exercise commitment.^96^

#### 3.4.2 Tele-rehabilitation (TR) and digital interventions

TR offers a viable solution to the logistical constraints and perceived burden associated with frequent, in-person medical centre visits. TR interventions have demonstrated the ability to significantly improve clinical outcomes in AS patients compared to baseline. A randomised controlled study reported that TR significantly improved within-group differences for disease activity (p = 0.003), function (p = 0.029), and mobility (p = 0.001) in axSpA patients. This technology-assisted, home-based approach has been shown to improve fatigue, morning stiffness, physical function, spinal mobility, and aerobic capacity.^97^

However, the efficacy of TR must be evaluated against the "gold standard" of face-to-face (FEG) supervision. An eight-week randomised controlled trial comparing FEG clinical Pilates to TR (synchronous video conference) in axSpA patients (n = 20) found a critical fidelity gap.^40^ While all measurements significantly improved in the FEG group (p < 0.05), key outcome improvements in the TR group, such as the Ankylosing Spondylitis Performance Index (ASPI) for putting on socks test, Hospital Anxiety and Depression Scale (HADS) scores, and Assessment of SpondyloArthritis international Society-Health Index (ASAS-HI) score, were not statistically significant (p > 0.05).

This disparity suggests that, to achieve maximal therapeutic outcome, particularly for complex biomechanical corrections required for optimal posture and functional sparing, the subtle interactive and physical feedback provided by high-fidelity, in-person supervision remains critical. The diminished efficacy in the remote TR group indicates that the quality control and precision of biomechanical instruction may be compromised via video link, making FEG the standard of care when accessibility is not prohibitive, especially for complex or high-risk patients requiring strict postural guidance to mitigate kyphotic progression.^98^

In contrast, certain mind-body exercises delivered via online platforms show particular promise. A 12-week randomised controlled trial demonstrated that Baduanjin qigong (online exercise program) was superior to a standard home-exercise control group. The intervention group showed a significant difference in favour of BASMI (p = 0.00-0.04), chest expansion (p = 0.04), and fatigue levels (FSS, p = 0.01).^41^ This suggests that specific, lower-impact, mind-body modalities translate effectively into the remote environment for symptomatic relief and flexibility gains.^99^

## 4 Conclusion

Physical rehabilitation in Ankylosing Spondylitis is a cornerstone of orthopaedic management, offering measurable benefits in mobility, function, and quality of life. Supervised, multimodal programs—especially those incorporating aquatic therapy, resistance training, and mind-body modalities—demonstrate superior adherence and clinical outcomes.^100^ While current protocols improve bone mineral density and alleviate symptoms, they fall short in modulating pathological ankylosis, highlighting a need for optimised mechanical loading strategies.^101^ Telerehabilitation expands access but lacks the precision of in-person supervision for complex biomechanical corrections.^102^ Perioperative rehabilitation, including prehabilitation and HO prophylaxis, is essential for surgical candidates. Overall, high-fidelity rehabilitation delays surgical intervention and mitigates fracture risk.^62^

## Consent to participate

Not applicable.

## Consent to publish

Not applicable.

## Guardian/patient's consent

Not applicable.

## Availability of data and materials

All relevant data are included in the article.

## Author contribution statement

NJ: Conceptualisation, Methodology, Literature search, Data curation, Writing – original draft, Project coordination; SR: Literature search, Data curation, Writing – review & editing; ASS: Literature search, Data extraction, Writing – review & editing; AN: Data interpretation, Writing – review & editing; SM: Methodological support, Writing – review & editing; LS: Writing – review & editing; FM: Writing – review & editing, Critical revision of the manuscript; MJ: Conceptualisation, Supervision, Methodological oversight, Critical revision of the manuscript. All authors approved the final version.

## Ethical approval

This study complies with ethical standards.

## Ethical statement

This study complies with ethical standards.

## Credit author statement

NJ: Conceptualisation, Methodology, Literature search, Data curation, Writing – original draft, Project coordination; SR: Literature search, Data curation, Writing – review & editing; ASS: Literature search, Data extraction, Writing – review & editing; AN: Data interpretation, Writing – review & editing; SM: Methodological support, Writing – review & editing; LS: Writing – review & editing; FM: Writing – review & editing, Critical revision of the manuscript; MJ: Conceptualisation, Supervision, Methodological oversight, Critical revision of the manuscript. All authors approved the final version.

## Use of AI

Language editing was performed using Grammarly (Superhuman Platform Inc., San Francisco, CA, USA).

## Funding

The authors received no financial support for the research, authorship, and/or publication of this article.

## Declaration of competing interest

The authors declare that they have no competing interests in this article.
